# Novel *MTTP* Gene Mutation in a Case of Abetalipoproteinemia with Central Hypothyroidism

**DOI:** 10.4274/jcrpe.galenos.2020.2020.0015

**Published:** 2020-06-03

**Authors:** Sezer Acar

**Affiliations:** 1 Dr. Behçet Uz Children’s Hospital, Clinic of Pediatric Endocrinology, İzmir, Turkey

**Keywords:** Central hypothyroidism, euthyroid sick syndrome, abetalipoproteinemia

Dear Editor,

I read the article of Soylu Üstkoyuncu et al (1) entitled “Novel *MTTP* Gene Mutation in a Case of Abetalipoproteinemia with Central Hypothyroidism” in Journal of Clinical Research in Pediatric Endocrinology with great interest. The diagnosis of abetalipoproteinemia was suspected in a 9-month-old patient with chronic diarrhea, failure to thrive, decreased subcutaneous adipose tissue, acanthocytosis, elevated liver function test, and decreased triglyceride and total cholesterol levels. Genetic analysis revealed a novel disease-causing variant in *MTTP*. In addition, because of mild-low free T4, normal free T3 and normal thyroid stimulating hormone (TSH) findings, central hypothyroidism was considered and L-thyroxine (12.5 mcg/day) was started and increased to 37.5 mcg/day at follow-up. Other pituitary hormones and pituitary imaging have been reported to be normal.

Central hypothyroidism is the failure of thyroid hormone synthesis associated with insufficient TSH stimulation. This occurs due to anatomical or functional disorders in the pituitary (secondary hypothyroidism) or hypothalamus (tertiary hypothyroidism) (2). Low free T4 and low/normal TSH levels would indicate central hypothyroidism. However, in hypothalamic disorders, slightly elevated TSH levels may be detected, as in subclinical or mild primary hypothyroidism cases (2). In the presented case, low free T4 and normal TSH level were considered to indicate central hypothyroidism. However, lack of TSH suppresion during follow-up with (even increasing) L-thyroxine suggests that the diagnosis of central hypothyroidism is questionable (2). Alexopoulou et al (3) demonstrated that mean TSH levels in treated central hypothyroidism patients were 0.2±0.5 mU/mL. Furthermore, normal (presented as low) baseline and high stimulated levels of cortisol of this patient suggest that thyroid abnormality may be associated with critical disease, not central hypothyroidism. In addition, thyroid function test results similar to central hypothyroidism may be seen in severe disease states, but this condition usually returns to normal with the improvement of the clinical picture (4). The authors report that normal free T3 level in the patient helped ruling out euthyroid sick syndrome, however, it has also been shown that thyroid hormone measurement results in immunoassay methods commonly used in our country are less reliable due to assay interference (5). Liquid chromatography-mass spectrometer method has been reported to offer superior specificity over the immunoassays for determination of thyroid hormones (5). Finally, if a diagnosis of central hypothyroidism is being reported to be associated with abetalipoproteinemia, sequencing of candidate genes are needed as well.

In addition, serum phosphate level (3.15 mg/dL) was considered normal according to adult reference range. However, this level is low compared to the appropriate age group (3.8-6.5 mg/dL) and warrants further assessment.

In conclusion, with limited data presented, the diagnosis of central hypothyroidism is suspicious in this patient and it would have been more appropriate to monitor thyroid function tests without treatment. There are various adaptive changes (such as hypothalamus-pituitary suppression) to reduce metabolic rate in severe disease states, which makes it difficult to diagnose central hypothyroidism. Taking all together, thyroid function tests should not be evaluated in cases with severe illness unless there is a strong suspicion of hypothyroidism.
